# Developing a national code of ethics for pharmacists in Kuwait: Insights from a survey and a modified Delphi study

**DOI:** 10.1371/journal.pone.0326089

**Published:** 2025-07-01

**Authors:** Salah Waheedi, Razan Alfailakawi, Shuaa Al-Selili, Hawraa Ghadanfari, Asmaa Al-Haqan

**Affiliations:** 1 Department of Pharmacy Practice, College of Pharmacy, Kuwait University, Kuwait; 2 Ministry of Health, Jabriya, Kuwait; University of Turin, ITALY

## Abstract

**Background:**

Pharmacists play a crucial role in healthcare and are expected to uphold high ethical standards. Establishing a code of ethics for pharmacists is essential as it supports improved patient outcomes and increases trust in the healthcare system. Regulatory organizations worldwide have recognized the significance of such codes in guiding professional behaviour and ensuring quality care. However, a national code of ethics for pharmacists in Kuwait has not yet been published. The aim of this study was to propose a national code of ethics for pharmacists in Kuwait, using insights from a survey of practicing pharmacists and a modified Delphi study involving expert consensus.

**Methods:**

A mixed methodology was employed comprising a survey of practicing pharmacists to gather their views on the clarity and acceptance of the 14 FIP standards and a modified Delphi study involving a group of experts to finalize and formulate the national code of ethics for pharmacists in Kuwait. For the survey, all registered pharmacists practicing in Kuwait were invited to participate through the Kuwait Pharmaceutical Association. Data collection occurred between February and May 2019 via an electronic questionnaire. The Delphi study participants were recruited through purposive and snowball sampling, with data collection taking place between March and June 2023. The Delphi study required a 90% consensus threshold for standard inclusion.

**Results:**

A total of 304 pharmacists (7.6% response rate) participated in the survey. The median age was 32 years, with a gender distribution of 56.6% female. The majority (85%) reported Arabic as their first language, and 75% held a BPharm degree. Most participants found the standards clear and acceptable, with clarity percentages generally above 88% and acceptance percentages similarly high. Standards 3 (patient autonomy) and 11 (competency of support staff) had slightly lower ratings, indicating potential areas for improvement. Comments highlighted issues such as patient knowledge and the clarity of support staff roles. In the Delphi study, 30 experts participated in the first round, with standards needing a 90% consensus for inclusion. Seven standards reached consensus in the first round, four in the second, and four in the third. Standard 11 failed to achieve consensus and was removed. The iterative process ensured thorough evaluation and refinement of each standard based on expert feedback.

**Conclusion:**

This study proposed a national code of ethics for pharmacists in Kuwait based on a comprehensive mixed-methods approach. The survey indicated high clarity and acceptance of most FIP standards among pharmacists, though areas for improvement were identified. The Delphi study achieved consensus on 13 out of 14 standards. This proposal provides a foundation for regulators to establish a formal code of ethics, aiming to enhance professionalism and patient trust in the healthcare system.

## Introduction

Pharmacists play a critical role in the healthcare system, providing essential services that impact patient care and public health. As trusted healthcare professionals, they are expected to adhere to high ethical standards to ensure the delivery of quality care and to foster trust within the community they serve. The establishment of a national code of ethics is pivotal in guiding pharmacists’ professional conduct, delineating the standards of practice, and promoting ethical decision-making.

Pharmacy is a profession that requires practitioners to adhere to ethical and professional standards that go beyond what is legally mandated. As the role of pharmacists continues to evolve, it is important to reaffirm and publicly state the obligations that guide their practice. These obligations, based on moral principles and values, provide guidance in interactions with patients, other healthcare professionals, and society at large. Key standards include beneficence, autonomy, justice, confidentiality, and adherence to laws.

Globally, many countries have recognized the importance of a standardized code of ethics for pharmacists [1]. These codes serve as a framework to address ethical dilemmas, ensure consistency in practice, and enhance the overall quality of healthcare services [2]. Despite the global recognition and the existence of various international standards, Kuwait has yet to establish a formal national code of ethics for its pharmacists [3].

The absence of a nationally recognized ethical framework poses challenges for pharmacists in maintaining consistent ethical practices [4]. This gap underscores the need for a structured code of ethics tailored to the cultural, social, and professional context of the Kuwaiti healthcare system. The development of such a code is essential not only to support pharmacists in their professional roles but also to enhance patient outcomes and trust in the healthcare system [4]. Lawmakers in Kuwait recognize the importance of a code of ethics, as it is included in the Pharmacy Law that pharmacists should adhere to such a code. However, the law does not require any authoritative organization to produce one, highlighting the need for an official and comprehensive code of ethics [5]. The code of ethics serves as the basis for the disciplinary powers of regulatory bodies. This legal backing is crucial for enforcing ethical behaviour and maintaining public trust in the profession [6].

The International Pharmaceutical Federation (FIP) stresses the critical need for pharmacists’ associations in every country to develop or support the creation of an up-to-date Code of Ethics. This directive is part of a broader effort to ensure that pharmacists worldwide adhere to consistent ethical standards that reflect the evolving scope of their responsibilities within healthcare systems [6].

Ethical challenges in pharmacy practice necessitate enhanced training in ethics, teaching of professionalism, establishment of a code of ethics, and improvement of communication skills.

This study aims to propose a national code of ethics for pharmacists in Kuwait, informed by the perspectives practicing pharmacists and expert consensus. Employing a mixed-methods approach, this research gathered views from a broad spectrum of pharmacists through a survey and achieved expert agreement on ethical standards using a modified Delphi technique [7]. The study used the 14 FIP standards as the base for the ethical standards proposed [6]. By integrating these diverse viewpoints, the study strives to deliver a comprehensive and culturally relevant ethical framework. This framework can be adopted nationally, empowering pharmacists in Kuwait to navigate ethical dilemmas and consistently uphold the highest professional standards.

## Methods

A mixed methodology was employed, comprising a survey of practicing pharmacists to gather their views on the clarity and acceptance of the 14 FIP standards, and a modified Delphi study involving a group of experts to finalize and formulate the national code of ethics for pharmacists in Kuwait. The Delphi technique is a research method that collects and analyses expert opinions through multiple rounds, ensuring anonymity and providing feedback after each round, allowing experts to revise their opinions and reach a consensus.

For the survey, all registered pharmacists (totalling 4000) practicing in Kuwait met the inclusion criteria and were thus invited to participate in the study through the Kuwait Pharmaceutical Association. An electronic version of the questionnaire was created using Google Forms, and a link to the questionnaire was sent via email and WhatsApp along with the invitation. Data collection occurred between February and May 2019.

For the modified Delphi study, participants were recruited through purposive or judgment sampling, complemented by snowball sampling. This approach involved selecting an initial group of participants who then recommended or referred others, creating a rolling effect that expanded the sample size. Data collection for this phase took place between March and June 2023.

The questionnaire was designed to include a section for demographics and a section listing the 14 FIP ethical standards. These standards, published by the International Pharmaceutical Federation, were presented in both English and an Arabic translation obtained from the Jordan Pharmaceutical Association website. Under each standard, participants were provided with a 5-point Likert scale to rate clarity (from completely unclear to completely clear) and another scale to rate acceptance (from completely unacceptable to completely acceptable). Additionally, a free text box was provided under each standard, allowing participants to specify any issues they had regarding the Arabic translation/ the clarity and/or level of acceptance.

For the Delphi study, a standard needed to reach a 90% consensus threshold to determine whether it should be included in the final version or re-evaluated in a subsequent round. This threshold was decided by the research team. In addition to the 90% consensus, any comments made were carefully reviewed by the researchers, as they could be decisive for including the standard in the final version or for its re-evaluation in the next round.

For the first round of the Delphi study, the electronic questionnaire was completed by the 30 participating experts. The Delphi participants were asked to report their level of acceptance and clarity for both the English and Arabic versions of the 14 standards, and to specify any issues they had with the standards. This process was repeated for rounds 2 (28 responded) and 3 (27 responded), after incorporating the changes specified by the participants and removing the standards that reached the 90% consensus threshold.

To ensure the Delphi technique was effectively implemented, all participants were required to be involved in all rounds. Participants who did not respond in the initial round were not permitted to participate in subsequent rounds. Additionally, to facilitate the completion of this process, reminders were sent to the participants one week after each round.

Ethical approval for this study was obtained from Human Ethical Committee, Health Sciences Center, Kuwait University 14/2019 and 270/2023.

Data were analysed using the Statistical Package for the Social Sciences (IBM SPSS Statistics, Version 29), allowing for a comprehensive interpretation of the gathered information. This study employed descriptive analysis for both the survey and the Delphi study. For the Delphi study, a consensus threshold was defined as 90% or above of the combined percentages of both acceptance and clarity. The Likert scales for acceptance and clarity were merged to make the results more interpretable and actionable, and to address the low sample size, providing in-depth insights and a detailed understanding of the responses to each standard.

## Results

### Survey of practicing pharmacists of Kuwait

A total number of 304 pharmacists participated in this survey, that is 7.6% of the registered pharmacists in Kuwait. The median age of the participants was 32 years (IQR: 28.0–39.8). The gender distribution was predominantly female, with 172 participants (56.6%), while males accounted for 102 participants (33.6%). For other characteristics, see Table 1.

**Table 1:** Demographic characteristics of survey participants.

Characteristics	N (%)
**Age:**	
Median age = 32	
IQR: 28.0–39.8	
**Gender:**	
Female	172 (56.6%)
Male	102 (33.6%)
**First language:**	
Arabic	258 (85%)
English	13 (4.3%)
Others	2 (0.7%)
**Pharmacy degree:**	
BPharm	228 (75.0%)
Others	45 (14.8%)
**Where did you study BPharm:**	
Kuwait University	94 (31%)
Abroad	152 (50%)
**Practice site:**	
Government hospital	155 (51%)
Polyclinics	70 (23%)
Private pharmacy	48 (16%)
**Practice year group:**	
≤5 years	88 (29%)
6-15 years	101 (33%)
16-25 years	44 (14%)
≥ 26 years	71 (23%)

The responses of pharmacists who found each standard to be clear or unclear, and those who accepted or did not accept each standard are summarised in Table 2. A small percentage of respondents remained neutral or unclear on both clarity and acceptance across all standards.

**Table 2:** Percentage of survey participants for the clarity and acceptance of standards.

	Clarity (N, %)		Acceptance (N, %)	
Standards	Yes	No	Yes	No
1-Equity in Resource Allocation	270 (91.2%)	9 (3.0%)	267 (91.8%)	7 (2.4%)
2-Patient Safety and Wellbeing	273 (92.9%)	7 (2.4%)	271 (93.4%)	4 (1.4%)
3-Patient Participation in Treatment Decisions	257 (88.3%)	8 (2.7%)	233 (80.9%)	18 (6.3%)
4-Collaboration with Health Professionals	276 (94.2%)	6 (2.0%)	269 (93.1%)	8 (2.8%)
5-Cultural Sensitivity in Patient Care	263 (89.8%)	7 (2.4%)	255 (89.2%)	9 (3.1%)
6-Confidentiality and Privacy	276 (94.5%)	5 (1.7%)	271 (93.8%)	4 (1.4%)
7-Adherence to Professional Standards	272 (93.2%)	7 (2.4%)	267 (93.0%)	4 (1.4%)
8-Integrity and Professional Conduct	281 (96.2%)	5 (1.7%)	274 (95.1%)	5 (1.7%)
9-Continuing Professional Development	274 (93.8%)	9 (3.1%)	264 (92.0%)	12 (4.2%)
10-Compliance with Legislation and Supply Chain Integrity	268 (91.8%)	7 (2.4%)	270 (93.4%)	6 (2.1%)
11-Competency of Support Staff	253 (86.6%)	18 (6.2%)	248 (86.1%)	16 (5.6%)
12-Accuracy and Objectivity of Information	278 (94.6%)	5 (1.7%)	276 (94.8%)	6 (2.1%)
13-Courtesy and Respect in Service	281 (95.6%)	6 (2.0%)	281 (96.9%)	2 (0.7%)
14-Continuity of Professional Services	262 (88.8%)	16 (5.4%)	254 (87.9%)	15 (5.2%)

A small percentage of respondents remained neutral or unclear on both clarity and acceptance across all standards

The majority of respondents found the standards to be clear and acceptable, with clarity percentages generally ranging above 88% and acceptance percentages also predominantly high.

Standard 3, which deals with patient participation in treatment decisions, and Standard 11, which deals with the competencies of support staff, had slightly lower clarity and acceptance rates compared to other standards, indicating potential areas for improvement.

Participant Comments on Standard 3:

- Patients lack the knowledge and may harm themselves.
- Patients read unreliable sources on the internet.
- Pharmacists are not part of the decision-making process in common practice in Kuwait.
- Patients do not prefer to be engaged in this decision.

Participant Comments on Standard 11:

- There is a shortage of pharmacists and professional technicians.
- Their [support staff] tasks are not clear.

It is noteworthy that no other comments or suggestions to change the Arabic translation were made by the participants throughout

### Three Delphi rounds

A total of 30 experts participated in the first Delphi round, 28 in the second, and 27 in the final round. Table 3 provides a detailed description of the demographics and professional characteristics of the participants. The participants had a median age of 36 years, with an interquartile range (IQR) of 32.3 to 40.5 years. The group was predominantly male, comprising two-thirds (66.7%, n = 20) of the participants. In terms of practice years, 30% (n = 9) had more than 15 years of practice experience. Among those with postgraduate qualifications, 26.7% (n = 8) had an MSc, and 13.3% (n = 4) had a PhD. Additionally, one participant held a Bachelor of Law alongside their pharmacy degree.

**Table 3:** Demographic and professional characteristics of the Delphi participants.

Characteristics	N (%)
**Age**	Median: 36 (IQR 32.3–40.5)
**Gender**	
- Female	10 (33.3%)
- Male	20 (66.7%)
**Nationality**	
- Kuwaiti	26 (86.7%)
- Non-Kuwaiti	4 (13.3%)
**Pharmacy Degree**	
- MPharm	5 (16.7%)
- BPharm	22 (73.3%)
- PharmD	3 (10%)
**BPharm Degree From**	
- Kuwait	10 (33.3%)
- Abroad	20 (66.7%)
**Postgraduate/Other Qualifications**	
- MSc	8 (26.7%)
- PhD	4 (13.3%)
- Bachelor of Law	1 (3.3%)
- No	17 (56.6%)
**Postgraduate Certificate From**	
- Kuwait	0 (0%)
- Abroad	11 (36.7%)
- No	19 (63.3%)
**Practice Site**	
- Administration Member	10 (33.3%)
- Member of Kuwait Pharmaceutical Association	5 (16.7%)
- Private Hospital	6 (20%)
- Public Hospital	12 (40%)
- Polyclinic	5 (16.7%)
**Practice Year Group**	
- Less than 5 years	5 (16.7%)
− 5–10 years	8 (26.7%)
− 11–15 years	8 (26.7%)
- More than 15 years	9 (30%)

**Fig 1:**
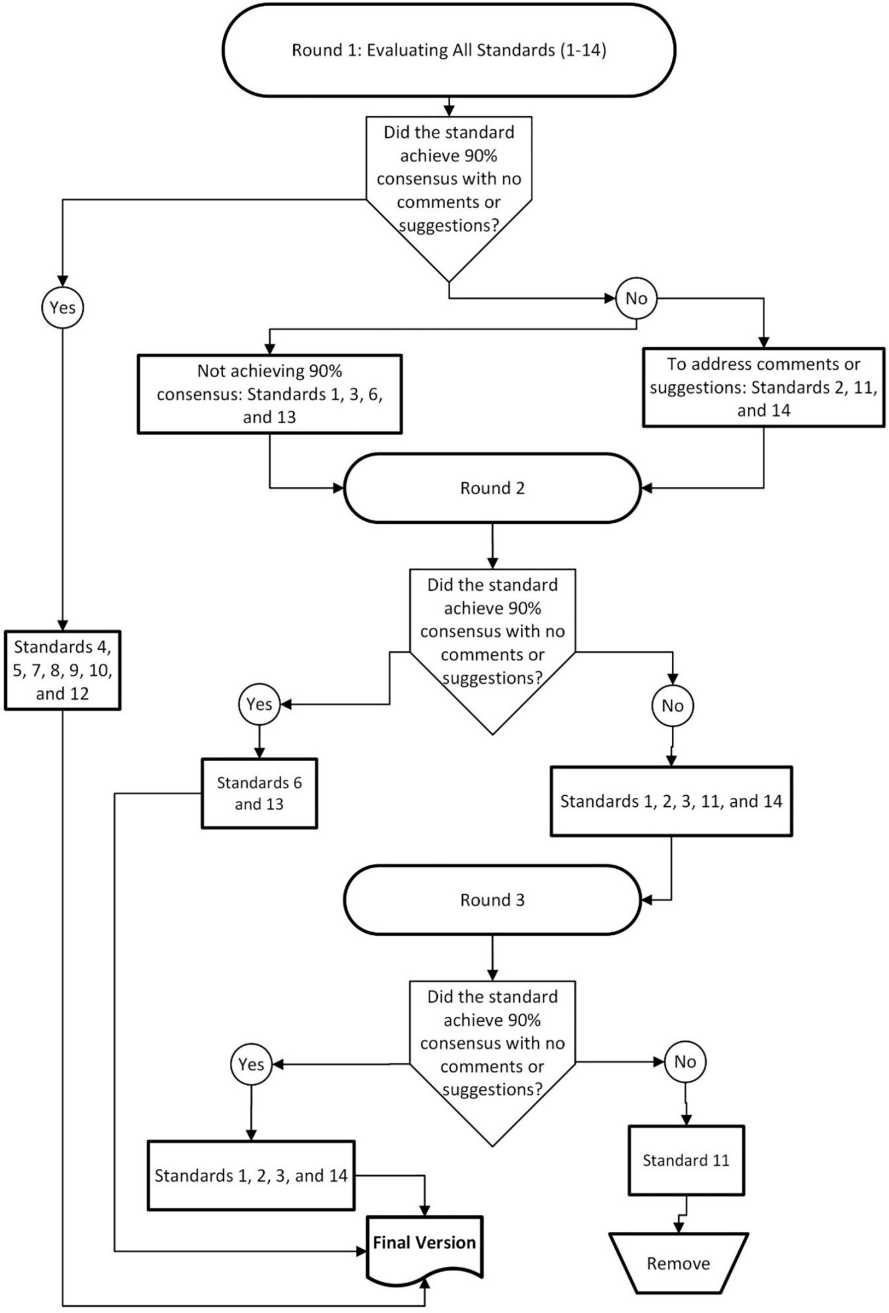
Summary of the three Delphi rounds. This is the Fig 1 legend.

In the first round, all 14 standards were evaluated by the 30 participants of the Delphi study who completed the online questionnaire. The detailed evaluation process is summarized in Fig 1. Standards 4, 5, 7, 8, 9, 10, and 12 achieved the threshold of 90% consensus with no comments or suggestions for changes to either the English or Arabic translations. As a result, these standards were finalized.

Standards 1, 3, 6, and 13 were questioned regarding their level of acceptance. For example, two participants marked Standard 1 as unacceptable. Therefore, comments for these standards were addressed, and they were sent to Round 2 for further evaluation.

Standards 2, 11, and 14 required a rephrased Arabic translation and further queries about their clarity and acceptance. Comments were addressed, and these standards were also sent to Round 2.

In Round 2, 28 out of the 30 participants responded to the online questionnaire. Standards 6 and 13 achieved the 90% consensus threshold with no additional comments or suggestions for changes. These standards were then finalized. However, Standards 1, 2, 3, 11, and 14 did not achieve the required consensus, particularly for the Arabic translations. Therefore, these standards were revised based on the feedback and sent to Round 3 for further evaluation.

During Round 3, 27 participants responded to the online questionnaire. Standards 1, 2, 3, and 14 achieved the 90% consensus threshold with no further comments or suggestions for changes. These standards were finalized. Standard 11, however, failed to achieve the required consensus and was subsequently removed from the final version.

## Discussion

The establishment of a national code of ethics for pharmacists in Kuwait is crucial for standardizing ethical practices and enhancing the quality of care provided to patients. Such a code serves as a fundamental guide for professional conduct, ensuring that pharmacists adhere to consistent ethical standards. This not only promotes trust within the community but also supports pharmacists in making informed and principled decisions. By providing clear guidelines, the national code helps navigate ethical dilemmas, ultimately fostering a more reliable and trustworthy healthcare system in Kuwait.

Integrating the FIP standards into the proposed code of ethics for pharmacists in Kuwait is a significant step in ensuring the framework is both robust and comprehensive. These standards, recognized globally, provide a solid foundation that aligns with international best practices. For example, the Pharmaceutical Society of Australia developed their Code of Conduct in 2017 based on similar principles, demonstrating the importance and effectiveness of such standards in guiding professional behaviour and ethical decision-making in the pharmacy profession [8]. By adopting these standards, the Kuwaiti code of ethics will benefit from established guidelines that enhance the quality and consistency of pharmacy practice.

Cultural considerations play a crucial role in shaping the ethical framework for pharmacists in Kuwait. The standard related to autonomy scored slightly lower than other standards in the survey and only achieved the 90% consensus threshold in the third Delphi round. This reflects the nuanced views on patient autonomy in Kuwait, where healthcare models may lean towards a more paternalistic approach compared to Western systems that emphasize patient autonomy. Family and community values significantly influence healthcare decisions, and patients often involve family members or religious leaders. Moreover, in Kuwaiti culture, patients may be less likely to question pharmacists’ recommendations due to respect to authority figures. Recognizing these cultural factors, the proposed code of ethics incorporates cultural competency training for pharmacists to better understand and respect patients’ perspectives on autonomy, thus facilitating informed decision-making while respecting cultural values.

Globally, codes of ethics for pharmacists often reflect their cultural and healthcare system differences. For instance, in the United States, the American Pharmacists Association Code of Ethics emphasizes individual patient rights and autonomy, which aligns with a predominantly patient-centered approach in healthcare [9]. Similarly, the United Kingdom’s General Pharmaceutical Council’s Standards for Pharmacy Professionals highlight patient-centered care, confidentiality, and respect for individual autonomy, reflecting its highly regulated and transparent healthcare system [10]. In contrast, many Asian countries, including India, maintain codes of ethics that blend patient care with traditional values of familial and communal decision-making [11]. This diversity in ethical standards highlights the need for cultural adaptability within the broader framework of professional ethics. By comparing Kuwait’s evolving code with these examples, we can identify unique challenges and opportunities to integrate global best practices while respecting local cultural norms.

Maintaining patient confidentiality is a cornerstone of ethical pharmacy practice which is emphasized by standard 6. To ensure its acceptance, it was suggested that exceptional cases to disclose patient information should be clearly specified. Participants also noted that patient consent to disclose information should not always be required in cases where public interest might be at stake. However, as with any standard, it is important to weigh the benefits and risks when deciding to uphold it. Balancing the need for confidentiality with public interest considerations is crucial to maintaining ethical standards in pharmacy practice.

In Japan, the Act on the Protection of Personal Information (APPI) regulates the handling of personal data. The 2022 amendment to the APPI has made the privacy law stricter, limiting the use of personal information outside of the intended and agreed purposes among providers [12]. These international perspectives provide valuable insights for Kuwait in defining its confidentiality standards within the broader ethical framework. In the European Union, the General Data Protection Regulation (GDPR) establishes strict guidelines for handling personal data, including health information. However, during public health emergencies, there is a need to balance individual privacy rights with public health interests. Ethical considerations of data sharing under the GDPR during the COVID-19 pandemic highlight that while the GDPR provides mechanisms for data processing in such crises, challenges remain in ensuring ethical and responsible data sharing. This includes considerations of data minimization, purpose limitation, and the necessity of implementing appropriate safeguards to protect individual privacy [13]. In China, the collection and processing of personal information during public health emergencies have evolved significantly. Initially, during the COVID-19 outbreak, there was widespread transmission of non-desensitized information in an unaccredited manner. Over time, China’s approach transitioned to a more orderly, legal, and effective system, optimizing the processing paths of personal information. This evolution reflects a balance between individual privacy rights and public health needs, aiming to protect public health while respecting personal information protection [14].

Standard 11 was removed due to its consistently low consensus percentage across the survey and Delphi rounds. The participants insisted on its removal, citing its ambiguity and impracticality given the diverse educational backgrounds and experience levels of technicians. They also raised concerns about the burden on pharmacists, resource constraints for training implementation, and potential duplication with existing regulatory standards. However, it could be argued that pharmacists should still be responsible for ensuring support staff competencies, providing necessary training, or appropriately tailoring their tasks.

Standard 14, which ensures continuity of professional services when pharmacists face conflicts with personal moral beliefs or pharmacy closures, was not accepted by all participants in the first two rounds. Participants found the standard incomprehensible and impractical, especially in the private sector, where pharmacists might face financial losses while continuing to provide care. Most pharmacists indicated they could provide services without conflicting with personal interests. Therefore, rephrasing the standard was found to be essential to avoid negative consequences. This perspective is supported by a study conducted by Esmalipour et al. (2021), which highlighted the ethical challenges faced by pharmacists in Saudi Arabia, including issues related to professionalism, communication, and regulatory policies [15].

The Delphi method proved effective in achieving consensus on the ethical standards for pharmacists in Kuwait, particularly by engaging a diverse group of experts and allowing for iterative feedback. One strength of this approach is its ability to integrate a wide range of perspectives, fostering a more comprehensive and culturally relevant code of ethics. However, the process of insisting on consensus, especially for fundamental principles, presented challenges. Defining consensus varied widely among studies, often lacking specific criteria or thresholds, which can impact the reliability of the results [7]. In our study, the criterion for consensus was set at 90% agreement, with the process continuing until this threshold was met. While this approach ensures robust agreement, it can also delay the conclusion of the study, as seen in the third round needed to achieve 90% consensus on the autonomy standard. Improved reporting of Delphi study methodologies, including clear definitions and stopping criteria, would enhance the reproducibility and transparency of such research efforts.

This study did not aim to investigate the ethical challenges faced in pharmacy practice directly, and there is a notable lack of literature on this topic specific to Kuwait. However, cross-sectional survey conducted in central Saudi Arabia provided valuable insights that could be applicable to the Kuwaiti context [16]. In Saudi Arabia, community pharmacists reported several barriers to solve ethical dilemmas effectively, such as lack of time, reliable resources, and knowledge on ethical matters. These challenges are likely similar in Kuwait and highlight the need for continuous ethics education and resource provision to support pharmacists in ethical decision-making.

The importance of ethics education in pharmacy cannot be overstated. Continuous ethics training and education for pharmacists are essential to prepare them for the complex ethical dilemmas they will encounter in their practice. Incorporating the proposed code of ethics into the educational curriculum for pharmacy students and professionals is crucial. This integration ensures that ethical considerations are embedded in their training from the outset, promoting a deeper understanding and commitment to ethical behaviour. Studies have shown that ethics education can significantly impact the attitudes and behaviours of healthcare professionals, leading to more ethical decision-making and improved patient care [15,16].

The regulatory landscape for pharmacists in Kuwait presents unique challenges, particularly because the Ministry of Health (MOH) is both the largest employer of pharmacists and the regulatory authority. This dual role can create conflicts of interest that complicate the implementation of a code of ethics. For example, in situations where there is a shortage of pharmacists or pharmacy technicians, the MOH might prioritize operational needs over strict adherence to competency standards. This could lead to a relaxation of requirements, undermining the ethical standards intended to ensure high-quality care.

Moreover, pharmacists might face ethical dilemmas where their obligation to patients conflicts with MOH policies or directives. In such cases, pharmacists might feel pressured to align with the ministry’s rules, potentially compromising patient care. This conflict can hinder pharmacists’ ability to put patients’ interests first, particularly if doing so would contradict the ministry’s regulations or orders.

Addressing these issues requires a clear separation of regulatory and employer roles to ensure unbiased enforcement of ethical standards. It also necessitates robust support systems for pharmacists, enabling them to navigate ethical dilemmas without fear of repercussions. By reinforcing the independence of regulatory oversight and providing continuous ethics education, the integrity of pharmacy practice can be upheld, ensuring that patient care remains the foremost priority.

### Implementation and future research

For the successful implementation of the proposed national code of ethics for pharmacists in Kuwait, the Kuwait Pharmaceutical Association (KPA) has adopted our proposal and submitted it to the Ministry of Health (MOH) for approval. This is a crucial step towards institutionalizing the ethical standards and ensuring they are integrated into pharmacy practice across the country. However, the approval and subsequent enforcement by the MOH are essential to translate these standards into practice.

This research represents the first step in introducing a comprehensive code of ethics for pharmacists in Kuwait. Future research and action plans will aim to broaden the scope by involving a larger and more representative sample of pharmacists to ensure inclusivity and diverse perspectives. Additionally, relevant ministries and institutions will be engaged as active participants to enhance collaboration and address potential challenges in implementation. These efforts will pave the way for a more robust and inclusive ethical framework for pharmacy practice in Kuwait.

Future research should focus on evaluating the impact of the implemented code on pharmacy practice and patient outcomes. Longitudinal studies could provide insights into how adherence to the code influences the quality of care, professional behavior, and public trust in pharmacists. Additionally, research should explore the barriers and facilitators to implementing the code, particularly in the context of the MOH’s dual role as both employer and regulator.

Further studies should also consider developing strategies to support pharmacists in navigating ethical dilemmas, ensuring that patient care remains the top priority even in challenging situations. This includes continuous ethics education and robust support systems that empower pharmacists to make ethical decisions confidently.

By conducting such research, we can ensure the proposed code of ethics is not only adopted but also effectively implemented, leading to a sustained improvement in ethical pharmacy practice in Kuwait.

### Limitations of the study

This study has several limitations that need to be acknowledged. First the response rate was lower than anticipated, which may impact the generalizability of the findings. Although all registered pharmacists in Kuwait were invited to participate in the survey, no specific sampling method was applied. This may have introduced nonresponse bias, as those who participated may have had stronger opinions or greater interest in the topic. Future research should explore strategies such as direct outreach, institutional collaborations, and incentives to improve response rates and ensure broader representation.

Additionally, the design of a lengthy questionnaire may have contributed to participant fatigue, potentially leading to incomplete or less accurate responses. This could affect the overall quality and reliability of the findings. Furthermore, the Delphi technique, while valuable for achieving expert consensus, has inherent challenges. As mentioned earlier, the insistence on reaching consensus for important principles can be challenging. The process can be time-consuming and demanding, leading to participant dropout, which may affect the representation and validity of the results.

Another limitation of the Delphi method is the potential for response bias., Participants might adjust their responses based on the feedback received in previous rounds, sometimes leading to a convergence on a compromise rather than a true consensus. Additionally, the selection of experts may introduce bias, as their perspectives and experiences can influence the final outcomes. Future studies should consider alternative approaches or complementary methodologies to mitigate these limitations and enhance the robustness of the findings.

## Conclusion

This study proposed a national code of ethics for pharmacists in Kuwait using a comprehensive mixed-methods approach, including a survey of practicing pharmacists and a modified Delphi study to achieve expert consensus. The survey indicated high clarity and acceptance of most of the 14 FIP standards among pharmacists, while the Delphi study refined these standards, achieving consensus on 13 out of 14. This process highlighted areas for improvement and the complexities involved in defining competencies for support staff.

The adoption of the proposed code by the Kuwait Pharmaceutical Association and its submission to the Ministry of Health marks a significant step towards institutionalizing these ethical standards. Further research should focus on evaluating the impact of the code on pharmacy practice and patient outcomes, addressing implementation challenges, and ensuring continuous ethics education for pharmacists. By effectively implementing this code, the pharmacy profession in Kuwait can enhance professionalism, improve patient trust, and ensure high-quality care.

## Supporting information

S1 Copy of Ethics in Kuwait data v2. (XLSX)
